# Genetic polymorphisms of *TRAPPC9* and *CD4* genes and their association with milk production and mastitis resistance phenotypic traits in Chinese Holstein

**DOI:** 10.3389/fvets.2022.1008497

**Published:** 2022-09-23

**Authors:** Muhammad Zahoor Khan, Gerile Dari, Adnan Khan, Ying Yu

**Affiliations:** ^1^Key Laboratory of Animal Genetics, Breeding, and Reproduction, Ministry of Agriculture and National Engineering Laboratory for Animal Breeding, College of Animal Science and Technology, China Agricultural University, Beijing, China; ^2^Faculty of Veterinary and Animal Sciences, Department of Animal Breeding and Genetics, The University of Agriculture, Dera Ismail Khan, Pakistan; ^3^Genome Analysis Laboratory of the Ministry of Agriculture, Agricultural Genomics Institute at Shenzhen, Chinese Academy of Agricultural Sciences, Shenzhen, China

**Keywords:** Chinese Holstein, milk protein, mastitis, SNP, CD4, *TRAPPC9*

## Abstract

The present study was designed to evaluate the association of polymorphisms in bovine *trafficking protein particle complex subunit 9* (*TRAPPC9)* and *cluster of differentiation 4* (*CD4)* genes with milk production and mastitis resistance phenotypic traits in a different cattle population. Three single nucleotide polymorphisms (SNPs) (SNP1 Position: Chr14:2484891, SNP2 (rs110017379), SNP3 Position: Chr14:2525852) in bovine *TRAPPC9* and one SNP (Position: Chr5:104010752) in *CD4* were screened through Chinese Cow's SNPs Chip-I (CCSC-I) and genotyped in a population of 312 Chinese Holsteins (156: Mastitis, 156: Healthy). The results were analyzed using the general linear model in SAS 9.4. Our analysis revealed that milk protein percentage, somatic cell count (SCC), somatic cell score (SCS), serum cytokines interleukin 6 (IL-6) and interferon-gamma (IFN-γ) were significantly (*P* < 0.05) associated with at least one or more identified SNPs of *TRAPPC9* and *CD4* genes. Furthermore, the expression status of SNPs in CD4 and *TRAPPC9* genes were verified through RT-qPCR. The expression analysis showed that genotypes GG in SNP3 of *TRAPPC9* and TT genotype in SNP4 of *CD4* showed higher expression level compared to other genotypes. The GG genotype in SNP2 and TT genotype in SNP3 of *TRAPPC9* were associated with higher bovine milk SCC and lower IL6. Altogether, our findings suggested that the SNPs of *TRAPPC9* and *CD4* genes could be useful genetic markers in selection for milk protein improvement and mastitis resistance phenotypic traits in dairy cattle. The CCSC-I used in current study is proposed to be validate in different and large population of dairy cattle not only in China but also in other countries. Moreover, our analyses recommended that besides SCC and SCS, the association of genetic markers could also be considered with the serum cytokines (IL-6, IFN-γ) while selecting genetically mastitis resistance dairy cattle.

## Introduction

Bovine mastitis is the inflammation of udder tissues with a marked decrease in milk quantity and quality in dairy cattle (1, 2). Bovine mastitis is one of the most costly diseases affecting dairy cattle's health and welfare globally (3, 4). This disease caused around $2 billion in losses to the US dairy industry annually (5). Because of mammary gland inflammation, the polymorphonuclear leukocytes from blood rush toward the site and results in a marked increase in milk's somatic cells content (6).

The somatic cell count (SCC) and somatic cell score (SCS) are the key indicators for susceptibility and resistance of a cow to mastitis (7–10). Due to the positive genetic correlation (0.4–0.8) between mastitis and SCC or SCS (11, 12), the strategy to minimize the risk of this disease by selecting dairy cattle against higher SCS is a worthy approach. However, the SCC and SCS are not constant and are influenced by many environmental factors (13, 14); therefore, in current research, we targeted serum cytokines (IL-6 and IFN-γ) in combination with SCC and SCS as mastitis resistance phenotypic traits.

Cytokines have dual nature, i.e., either activate or repress the inflammatory response and thus play a vital role in mastitis development (15). In addition, the increased levels of IFN-γ, IL-6 and IL-17 have been documented in acute mastitis (16). Consistently, a study showed that the detection of IL-6 in milk indicated subclinical mastitis earlier than SCC (17). Although the mentioned phenotypic traits are good indicators of mastitis, however, due to low heritability, mastitis resistance will yet be a challenge for animal breeders (18). Therefore, the association of mastitis resistance phenotypic traits with a polymorphism in genes is the research of interest in the modern dairy industry for control of mastitis.

The researchers rely on a marker-assisted selection strategy for mastitis resistance (19, 20). The candidate gene approach, which takes in account of SNPs in the genes that are associated with these traits, is a widely used method to control mastitis (19). Being quantitative traits, mastitis and milk production traits are controlled by many genes (21). The *TRAPPC9* and *CD4* are the key genes that play an important role in developing innate immunity and milk production traits.

The *TRAPPC9* gene, residing on bovine chromosome 14 is the vital member of the nuclear factor kappa B (NF-κB) family which has an essential role in inflammation and innate immunity (22–24). The elevated level of *TRAPPC9* gene enhances the activity of NF-κB signaling during mastitis development in dairy cattle (22). The associations of polymorphisms in *CD4* and *TRAPPC9* genes with milk production and mastitis resistance traits have been documented in previous reports (2, 25–27). Similarly, our in our previous study by using transcriptomic screening, we reported that *TRAPPC9* gene was significantly associated with milk SCC and bovine mastitis susceptibility (28). Recently, a study have reported the association of *TRAPPC9* gene with milk fat and immunity in Ayrshire and Jersey dairy cattle (29). In addition, the increase in milk CD4+ T cells was documented to be correlated with non-specific mastitis which suggested their link with low bacterial shedding (30).

Keeping in view the importance of these two immunity-associated genes (*TRAPPC9, CD4*); we selected three polymorphisms (SNP1 Position: Chr14:2484891, SNP2 (rs110017379), SNP3 Position: Chr14:2525852) in bovine *TRAPPC9* and one SNP (Position: Chr5:104010752) in *CD4* from our previous studies and validate them in a new and large Chinese Holstein population. For this purpose, these SNPs in *TRAPPC9* and *CD4* genes were detected by a new technique, i.e., Chinese Cow's SNPs Chip-I (CCSC-I) and genotyped in a different and a bit larger Chinese Holsteins population to explore their association with mastitis resistance and milk production phenotypic traits.

## Materials and methods

### Ethical statement

All animal procedures were performed according to the regulation approved by the ethical committee of the College of Animal Science and Technology, China Agriculture University, Beijing, PR China [Permission number: DK996]. All the data was collected from China Agriculture University dairy farm and no consent was needed from farmers.

### Sample size and collection

We randomly selected a total of 312 Chinese Holstein cows (156: Mastitis, 156: Healthy) in parities ranging 1–3 from a single dairy farm in Beijing China. In addition, based on SCC level, the cows were confirmed as mastatic (cattle with SCC higher than 200,000/ml) or healthy (cattle with SCC lower than 200,000/ml). The blood samples were collected from the caudal vein of all the selected population of Chinese Holsteins in 9 mL of 3 tubes including one each for DNA extraction (EDTA coated tube), RNA extraction, and serum isolation (non-EDTA tube). For serum isolation, the blood samples were placed at room temperature for 30 min to enable blood coagulation and then centrifuged at 3,000 rpm for 10 min to separate serum. The serum samples were stored at 4°C and sent to the Beijing Huaying Biological Technology Research Institute within 24 h to detect the concentration of IL-6 and IFN-γ. The milk SCC data were obtained from the Beijing Jinyindao Dairy Farm data record section, while SCS was calculated using the formula: SCS = log2 (SCC / 100,000) + 3.

### DNA extraction, SNP identification and genotyping

Genomic DNA was isolated from blood samples of 312 Chinese Holstein using Tiangen Blood DNA Kit (Tiangen Biotech Co., China) following the manufacturer's instructions. The quantity and quality of DNA were measured using NanoDrop ND-2000c Spectrophotometer (Thermo Scientific, Chelmsford, MA, USA) and gel electrophoresis. After confirmation of quality and quantity, all the DNA samples were sent to Capital Bio Technology Co., Ltd, Beijing, China, for identification of SNPs and genotyping with Chinese Cow's SNPs Chip-I. The selected SNPs in *TRAPPC9* and *CD4* genes were genotyped in the different and bit large population of 312 Chinese Holstein.

### RNA isolation and purification

Total RNA extraction from the Holstein cattle's white blood cells was carried out through the standard TRIzol method (Invitrogen, Carlsbad, CA, USA) following the manufacturer's protocols. RNase-Free DNase Set (QIAGEN) was used to purify RNA and to ensure genomic DNA elimination. The quantity and quality of RNA were measured by using a NanoDropTM ND-2000c Spectrophotometer (Thermo Scientific, Inc.), and the integrity of RNA was monitored on 1% agarose gel.

### Reverse transcription and primer design

According to the manufacturer's instructions, reverse transcription was performed using PrimeScript 1st Strand cDNA Synthesis kit (TaKaRa, Dalian, China). The PCR primers for the bovine *CD4, TRAPPC9*, and a housekeeping gene *GAPDH* (glyceraldehyde-3-phosphate dehydrogenase) were designed by Primer-Blast on NCBI and synthesized by Beijing Genomics Institute Tech, based on the golden rules for real-time reverse transcription PCR (RT-PCR). The amplification efficiency of these primer pairs was tested by RT-qPCR initially, and the mRNA expression of the two genes was normalized against the housekeeping gene *GAPDH* by the cDNA in the corresponding samples. Three pairs of primers designed for *GAPDH, TRAPPC9*, and *CD4* are given in Table 1. For mRNA expression analysis, four samples for each SNP were run in triplicate.

**Table 1:** Detail of PCR primers of qRT-PCR used in the study.

**Primers**	**Sequence of primers**
*CD4*	F: 5'-CCACTGGGACCTGAGGTGTC-3'
	R: 5'-GCATCACCACACCAATTCA-3'
*TRAPPC9*	F: 5'-CTGCTCCGCTCGGTGAATGAC-3'
	R:5'- CGTTCTCTGCCTTGACTGTG-3'
*GAPDH*	F: 5'-CCCTGAGACAAGATGGTGAAG-3'
	R:5'-CATGTAGTGAAGGTCAATGAAG-3'

### Gene expression analysis by RT-QPCR

Real-time quantitative polymerase chain reaction (RT-qPCR) was performed to determine *TRAPPC9* and *CD4* genes expression levels. The reactions were performed in a total volume of 20 μL containing 2 μL cDNA, 1 μL each primer, 10 μL SYBR Green Master Mix (Roche, Penzberg, Germany), 6 μL nuclease-free water using the following amplification condition: 94°C for 10 min, followed by 44 cycles of 94°C for 15 s, 60°C for 10 s, 72°C for 10 s, and 72°C for 30 s. Fluorescence signals were collected at 60°C step. Mean was consequential from the two repeats for each sample. Light Cycler 480 RT-PCR system was used to perform amplification, detection and data analyses.

### Statistical analysis

The allele and genotype frequencies were tested for deviations from proportions of Hardy–Weinberg equilibrium (HWE) by using Chi-square test (χ2). The association analysis of SNPs in *TRAPPC9* and *CD4* with milk production and mastitis-related traits were carried out by the least-squares method as applied in the GLM procedure of SAS (SAS Institute Inc., Cary, NC, USA) according to the following linear model.


$$\begin{array}{l} P_{ijkn=}\mu +f_{i+}p_{j+}snp_{k+}e_{ijkn} \end{array}$$

where *P*_*ijkn*_ indicates mastitis traits (SCC, SCS or serum concentration of cytokine IL-6 and IFN-γ) or milk production traits (fat percentage or protein percentage), μ is overall mean, *f*_*i*_ is the fixed effect of the farm, *p*_*j*_ is the fixed effect of parity, *snp*_*k*_ is the fixed effect of genotype, *e*_*ijkn*_ is the random residual error.

The estimated genotype effects were further divided into additive effect (A) and dominant effect (D). The additive effect was the mean deviation of two homozygous genotypes (Formula 1), and the dominant influence was calculated by the deviation of the heterozygous genotype from the mean of two homozygous genotypes (Formula 2) (31).


$$\begin{array}{l} \;\text{A}=(\text{AA}-\text{BB})/2\;(\text{Formula}\;1)\\ \text{D}=\text{AB}-(\text{AA}+\text{BB})/2\;(\text{Formula}\;2) \end{array}$$

Where, AA, AB and BB were least square means of genotype AA, AB and BB, respectively.

Student *t*-test was performed for RT-qPCR analyses for the comparison of mRNA expression levels of different genotypes of SNPs in the two genes (*TRAPPC9* and *CD4*).

## Results

### The SNPs information, identification and genotyping

Three SNPs in *TRAPPC9* and one SNP from *CD4* gene were screened through CCSC-I (10) and genotyped in a total of 312 (156: Mastitis, 156: Healthy) Chinese Holstein population. It was found that except the SNP1, all SNPs' allele and genotypic frequencies were in Hardy–Weinberg equilibrium (*P* > 0.05). The observations, genotypic and allelic frequencies and values of Chi-square test (χ^2^) of the selected SNPs in the present study are summarized in Table 2.

**Table 2:** The information of single nucleotide polymorphisms and their Allelic and genotypic frequencies in *TRAPPC9* and *CD4* genes.

**SNP (Gene)**	**Mutation**	**Reference**	**Position**	**Genotypes**	**Allelic frequency**	**Genotypic frequency**
SNP1	C-T/Exon2	Novel	Chr14:2484891	CC(303)	0.974277	C(0.98)
*(TRAPPC9)*				CT(3)	0.009646	T(0.02)
				TC(5)	0.016077	
SNP2	A/G	rs110017379	Chr14: 2607583	AA(122)	0.391026	A(0.62)
*(TRAPPC9)*				AG(144)	0.461538	G(0.38)
				GG (46)	0.147436	
SNP3	T-G/Intron6	Novel	Chr14:2525852	TT(64)	0.205128	T(0.46)
*(TRAPPC9)*				TG(158)	0.50641	G(0.54)
				GG(90)	0.288462	
SNP4	C/T Promoter	Novel	Chr5:104010752	CC(148)	0.48	C(0.70)
*(CD4)*				CT(141)	0.45	T(0.30)
				TT(23)	0.07	

### Association of mutations in TRAPPC9 and CD4 genes with milk production and mastitis resistance phenotypic traits

The association of three SNPs in *TRAPPC9* was evaluated with milk production and mastitis resistance traits. Our findings illustrated that SNP1 was significantly associated with milk protein, the SNP2 at position A/G 2607583 linked notably with SCS (*P* < 0.05), whereas the SNP3 (T/G 2525852) with SCC, SCS, and serum cytokine IL-6 (*P* < 0.05). Additionally, the association analysis revealed that the SNP in the *CD4* gene (104010752C/T) did not show any link with milk production traits, however, revealed a significant association with IFN-γ and IL-6 (*P* < 0.05). Finally, the association analysis showed that the genotypes GG in SNP3 and homozygous TT (SNP4) were significantly associated with low SCC, SCS and a higher level of IL-6 (Table 3). Similarly, the analyses revealed that genotypes TT (SNP3) and CC (SNP4) were associated with low IL-6 and high SCC level, which make the dairy cattle more vulnerable to the mastitis development.

**Table 3:** Association of SNPs in *TRAPPC9* and *CD4* genes on SCC, SCS, milk production, and serum cytokines traits in Chinese Holsteins.

**SNP**	**Genotype**	**Fat (%)**	**Protein (%)**	**IL-6**	**IFN-γ**	**SCC**	**SCS**
SNP1	CC(303)	4.09 ± 0.05	2.93 ± 0.15	67.19 ± 1.43	96.43 ± 1.12	330.98 ± 39.93	2.60 ± 0.15
(*TRAPPC9*)	CT(3)	4.43 ± 0.53	3.82 ± 0.18	57.58 ± 16.83	107.23 ± 13.22	40.66 ± 400.05	1.69 ± 1.46
	TC(5)	4.44 ± 0.41	2.91 ± 0.15	77.18 ± 11.90	100.71 ± 9.35	162.2 ± 309.87	2.80 ± 1.13
	*P-value*	0.57	**<** 0.0001^***^	0.59	0.65	0.67	0.81
SNP2	AA(122)	4.08 ± 0.08	3.05 ± 0.03	70.03 ± 2.23	95.28 ± 1.75	219.87 ± 62.23	2.08 ± 0.23
(*TRAPPC9*)	AG(144)	4.12 ± 0.07	3.06 ± 0.02	65.62 ± 2.08	98.65 ± 1.64	375.36 ± 57.68	2.87 ± 0.21
	GG (46)	4.08 ± 0.13	3.04 ± 0.05	64.70 ± 2.66	94.21 ± 2.88	444.8 ± 101.34	3.05 ± 0.37
	*P-value*	0.89	0.87	0.26	0.24	0.08	0.01^**^
SNP3	TT(64)	4.16 ± 0.12	3.04 ± 0.04	65.08 ± 3.09	96.78 ± 2.44	515.68 ± 85.52	2.91 ± 0.31
(*TRAPPC9*)	TG(158)	4.14 ± 0.07	3.08 ± 0.03	64.85 ± 1.96	97.88 ± 1.55	316.07 ± 54.50	2.83 ± 0.20
	GG(90)	4.99 ± 0.10	3.03 ± 0.04	72.96 ± 2.59	94.43 ± 2.05	201.79 ± 74.52	1.92 ± 0.26
	*P-value*	0.41	0.48	0.03^**^	0.41	0.02^**^	0.01^**^
SNP4	CC(148)	4.07 ± 0.08	3.07 ± 0.03	63.14 ± 2.01	98.33 ± 1.59	294.85 ± 56.97	2.79 ± 0.21
(*CD4*)	CT(141)	4.12 ± 0.08	3.03 ± 0.03	68.64 ± 2.02	96.53 ± 1.62	376.8 ± 58.98	2.51 ± 0.21
	TT(23)	4.19 ± 0.19	3.15 ± 0.02	85.32 ± 5.17	86.18 ± 4.14	195.26 ± 144.03	1.75 ± 0.52
	*P-value*	0.82	0.23	0.0003^***^	0.02^**^	0.39	0.17

P-value is the significance level from analyses of the association of SNPs with milk production, SCC, SCS, and serum cytokines traits in Chinese Holsteins

(^***^P < 0.001;

** P < 0.01;

^*^P < 0.05).

### Additive and dominant effect of polymorphism in *TRAPPC9* and CD4 genes on milk production and mastitis resistance phenotypic traits

The additive and dominant effect of SNPs in *CD4* and *TRAPPC9* genes are summarized in Table 4. The current association analysis for the additive and dominant effect of polymorphisms (SNP1, SNP2, SNP3and SNP4) revealed that dominant effect of SNP1 is significantly (*P* < 0.05) associated with milk protein, whereas SNP3 showed a significant additive effect on SCC, SCS and IL-6 (*P* < 0.05). Similarly, the additive effect of SNP2 was significantly associated with SCS and the SNP4 in *CD4* was correlated with IFN-γ and IL-6 (*P* < 0.05) (Table 4).

**Table 4:** Genetic effect of SNPs in *TRAPPC9* and *CD4* genes on milk production, mastitis resistance and serum cytokine traits in Chinese Holstein.

**SNPs**	**Effect**	**Fat %**	**Protein %**	**IL-6**	**IFN-γ**	**SCC**	**SCS**
SNP1	A	−0.18 ± 0.21	0.07 ± 0.07	−4.99 ± 5.99	−2.14 ± 4.71	84.39 ± 156.21	−0.09 ± 0.57
	*P-value*	0.39	0.35	0.41	0.64	0.58	0.86
	D	0.16 ± 0.56	1.15 ± 0.20	−14.61 ± 17.87	8.67 ± 14.03	−205.92 ± 429.46	−1.009 ± 1.57
	*P-value*	0.78	<0.0001^***^	0.41	0.53	0.68	0.52
SNP2	A	0.00 ± 0.08	0.003 ± 0.03	2.66 ± 2.14	0.54 ± 1.69	−112.45 ± 59.45	−0.48 ± 0.22
	*P-value*	0.99	0.91	0.21	0.75	0.06	0.02^**^
	D	0.04 ± 0.11	0.02 ± 0.04	−1.74 ± 2.99	3.91 ± 2.35	46.83 ± 82.83	0.30 ± 0.30
	*P-value*	0.66	0.61	0.56	0.99	0.61	0.32
SNP3	A	0.08 ± 0.08	0.01 ± 0.03	−3.94 ± 2.01	1.17 ± 1.59	156.95 ± 56.06	0.50 ± 0.20
	*P-value*	0.26	0.86	**0.05** ^*^	0.46	**0.005** ^***^	**0.02** ^**^
	D	0.07 ± 0.10	0.05 ± 0.04	−4.17 ± 2.81	2.29 ± 2.22	−42.66 ± 78.26	0.42 ± 0.29
	*P-value*	0.53	0.25	0.13	0.31	0.58	0.14
SNP4	A	−0.06 ± 0.10	−0.04 ± 0.04	−11.08 ± 2.77	6.07 ± 2.21	49.79 ± 77.44	0.52 ± 0.28
	*P-value*	0.58	0.31	**<0.0001** ^***^	0.006^***^	0.52	0.06
	D	−0.01 ± 0.13	−0.082 ± 0.048	−5.59 ± 3.43	4.27 ± 2.75	131.74 ± 96.98	0.25 ± 0.35
	*P-value*	0.92	0.08	0.104	0.12	0.17	0.49

A means additive effect, D means dominant effect, single asterisk

(*) shows that the additive and dominance effect of the locus is significant (P < 0.05), while

*** P < 0.001 and

** P < 0.01

demonstrate that the additive and dominance effect of the locus is highly significant.

### The MRNA relative expression assays of genotypes of SNPs in *TRAPPC9* and CD4 genes

**Figure 1:**
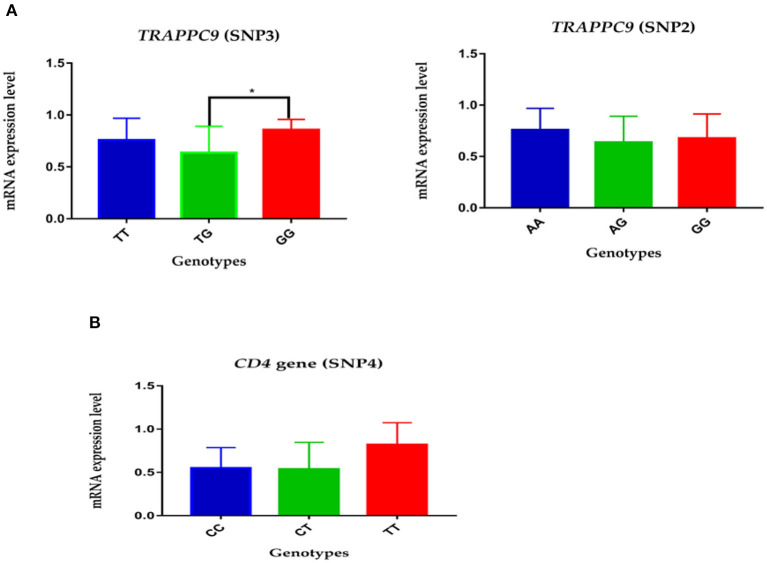
The relative mRNA expression level of polymorphisms in *TRAPPC9* and *CD4* gene: **(A)** The expression level of genotypes in SNP2 and SNP3 of *TRAPPC9:* the GG genotype show significantly higher relative mRNA expression than TG in SNP3; however, no significant difference was reported among the three genotypes of SNP2. **(B)** The relative genotypes expression of SNP4 in *CD4* gene: the TT genotype showed comparatively higher relative mRNA expression than CT and CC.

The expression level of mutations in the *TRAPPC9* and *CD4* genes was measured by real-time quantitative PCR. The analysis showed that the relative mRNA expression of AA genotype in SNP2 had higher expression compared to GG and AG genotypes in the *TRAPPC9* gene. Similarly, the GG genotype in SNP3 in *TRAPPC9* demonstrated significantly higher expression than TG genotype in the given Chinese Holsteins dairy cattle population (Figure 1A, *P* < 0.05). Moreover, the genotype TT's mRNA expression level was comparatively higher than CC and CT genotypes in the SNP4 of *CD4* gene (Figure 1B).

## Discussion

Recently, single nucleotide polymorphisms in many genes have been found to be associated with milk production and mastitis resistance traits suggesting that these variants could be used as potential genetic markers in modern breeding schemes for the improvement of production and increasing resistance to mastitis. In the present study, the polymorphisms in *TRAPPC9* and *CD4* genes that cause variation in the economic and health traits (milk production and mastitis resistance phenotypic traits) were selected from our previous studies and analyzed for validation in a new and larger population by using CCSC-I. To our knowledge, this is the first study in which we practically applied CCSC-I in mastitis resistance research. Bovine SNP Chip's application was previously used by Mullen and his co-workers in 2013 for dairy and beef production research (32). Similarly, a research study has also reported that SNP Chip is a cost and time-effective approach for implementing genomic selection in livestock (33). Keeping in view the importance of the SNP Chip from various published studies we used this technique in the present study to validate the role of the significant variants from our previous studies in *CD4* and *TRAPPC9* genes for production and mastitis resistance traits. Our research team reported in previous studies that SNPs at position 2484891 C/T, 2525852 T/G and 2607583 A/G in *TRAPPC9* were associated with milk protein and fat percentages (34). In contrast, polymorphism at position 2525852 T/G (*TRAPPC9*) did not show any link with milk contents (35). Furthermore, the mutation (2607583 A/G) was noticed to be associated with SCS, while the SNPs at position 2484891 C/T, and 2525852 T/G were linked to IL-6 and IFN-γ, respectively, however no correlation of 2607583 A/G in *TRAPPC9* gene was found with SCC, SCS and serum cytokines (IL-6 and IFN-γ) (36). In comparison, we found that the SNP (2484891 C/T) was linked to protein percentage, whereas the polymorphisms 2525852 T/G and 2607583 A/G were associated with IL-6, SCS, SCC, and SCS, respectively. Moreover, the mutation at point C104010752T was significantly correlated to milk SCC in Chinese Holsteins (26) and mutation at point g.13598C>T was linked to milk yield, protein and SCS (27). Importantly, in the new dairy population, our results further revealed the noteworthy correlation of SNP (C104010752T) with IL-6 and IFN-γ instead of SCC and SCS (*P* < 0.05). Based on our current findings, we reported that the SNPs in *TRAPPC9* and *CD4* genes show pleiotropic ability; however, it is also possible that the documented polymorphisms in the current study and our previous research might be influenced by population size and environmental factors.

Within an SNP, allele combinations and genotypes exert a critical role in the regulation of any traits. In the current study, we found that the homozygous GG genotype in SNP3 (*TRAPPC9*) and TT genotype in SNP4 (*CD4*) were associated with a higher level of IL-6 and a low level of SCC. These findings suggested that the mentioned genotypes (GG and TT) are linked with mastitis resistance and should be considered as potential markers while selecting genetically mastitis resistance cattle. Finally, the primary functional validation of SNPs in *TRAPPC9* and *CD4* genes were verified through RT-qPCR. Similar trends for all the genotypes in SNPs (*TRAPPC9* and *CD4*) that were found for their association with serum cytokines and mastitis resistance phenotypic traits were also reported in expression analysis. In relation to the association of the genetic variants to the specific mastitis resistance phenotypic trait and the level of significance documented in our previous studies, there were some differences in the present study's findings and in the previous studies, which still need further validation in large and different population of Chinese Holsteins.

In general, the present study using a newly designed CCSC-I for genotyping to validate the association of SNPs in *TRAPPC9* and *CD4* genes with milk production and mastitis resistance traits. Although we reported the significant link of the selected SNPs in *TRAPPC9* and *CD4* genes milk production and mastitis resistance phenotypic traits, however, we recommend further in-depth studies to test the documented reported SNPs of *TRAPPC9* and *CD4* in a large Chinese Holstein population as well as in other different dairy breeds from various regions of the world by using our newly designed CCSC-I for the validation of its capability to improve milk production and mastitis resistance in dairy cattle.

## Conclusions

Overall, the present study validated the three SNPs of *TRAPPC9* and *one* SNP of *CD4* in a large and different Chinese Holstein population by using our newly designed CCSC-I. The results verify that the documented SNPs in both genes (*TRAPPC9* and *CD4)* could be considered as powerful genetic markers against bovine mastitis resistance. The targeted SNPs in the *TRAPPC9* gene might be used as a marker for improved milk protein percentage as well. The study proposed that the CCSC-I could also be validated in more large dairy cattle population not only in China but also in other countries across the globe. Additionally, the upshot of a study infers that not only SCS and SCC but IL-6 and IFN-γ association can be establish with genetic markers while selecting genetically mastitis resistance dairy cattle.

## Data availability statement

The original contributions presented in the study are included in the article/supplementary material, further inquiries can be directed to the corresponding author/s.

## Ethics statement

The animal study was reviewed and approved by the Ethical Committee of the College of Animal Science and Technology, China Agriculture University, Beijing, PR China [Permission number: DK996].

## Author contributions

Conceptualization and methodology: MK and YY. Validation: MK. Resources: MK and GD. Writing—original draft preparation: MK. Writing—review and editing: MK, AK, YY, and GD. Supervision: YY. All authors have read and agreed to the published version of the manuscript.

## Funding

This article was financially supported by the National Key R&D Program of China (2021YFD1200900, 2021YFD1200903), NSFC-PSF Joint Project (31961143009), Beijing Dairy Industry Innovation Team (BAIC06), China Agriculture Research System of MOF and MARA, Beijing Natural Science Foundation (6182021), and the Program for Changjiang Scholar and Innovation Research Team in University (IRT-15R62).The funders had no role in study design, data collection, and analysis, decision to publish, or preparation of the manuscript.

## Conflict of interest

The authors declare that the research was conducted in the absence of any commercial or financial relationships that could be construed as a potential conflict of interest.

## Publisher's note

All claims expressed in this article are solely those of the authors and do not necessarily represent those of their affiliated organizations, or those of the publisher, the editors and the reviewers. Any product that may be evaluated in this article, or claim that may be made by its manufacturer, is not guaranteed or endorsed by the publisher.
